# *In Situ* and *Ex Situ* TEM Study of Lithiation Behaviours of Porous Silicon Nanostructures

**DOI:** 10.1038/srep31334

**Published:** 2016-08-30

**Authors:** Chenfei Shen, Mingyuan Ge, Langli Luo, Xin Fang, Yihang Liu, Anyi Zhang, Jiepeng Rong, Chongmin Wang, Chongwu Zhou

**Affiliations:** 1 Mork Family Department of Chemical Engineering and Materials Science, University of Southern California, Los Angeles, California 90089, United States; 2 National Synchrotron Light Source II, Brookhaven National Laboratory, Upton, New York 11973, United States; 3 Environmental Molecular Sciences Laboratory, Pacific Northwest National Laboratory, Richland, Washington 99352, United States; 4 Ming Hsieh Department of Electrical Engineering, University of Southern California, Los Angeles, California 90089, United States

## Abstract

In this work, we study the lithiation behaviours of both porous silicon (Si) nanoparticles and porous Si nanowires by *in situ* and *ex situ* transmission electron microscopy (TEM) and compare them with solid Si nanoparticles and nanowires. The *in situ* TEM observation reveals that the critical fracture diameter of porous Si particles reaches up to 1.52 μm, which is much larger than the previously reported 150 nm for crystalline Si nanoparticles and 870 nm for amorphous Si nanoparticles. After full lithiation, solid Si nanoparticles and nanowires transform to crystalline Li_15_Si_4_ phase while porous Si nanoparticles and nanowires transform to amorphous Li_x_Si phase, which is due to the effect of domain size on the stability of Li_15_Si_4_ as revealed by the first-principle molecular dynamic simulation. *Ex situ* TEM characterization is conducted to further investigate the structural evolution of porous and solid Si nanoparticles during the cycling process, which confirms that the porous Si nanoparticles exhibit better capability to suppress pore evolution than solid Si nanoparticles. The investigation of structural evolution and phase transition of porous Si nanoparticles and nanowires during the lithiation process reveal that they are more desirable as lithium-ion battery anode materials than solid Si nanoparticles and nanowires.

With ever-growing demands for high-performance power sources, especially in portable electronics and electrical vehicles (EV), tremendous research interests have been stimulated toward developing the next generation of lithium-ion batteries (LIBs) with high capacity, long cycle life, and low cost^1^,^2^. Compared with carbonaceous anodes (372 mAh/g for LiC_6_) used in commercial LIBs, silicon (Si) has a large theoretical gravimetric capacity of ~4200 mAh/g and volumetric capacity of ~8500 mAh/cm^3^, and therefore has been considered as one of the most promising anode materials for the next-generation LIBs^3^,^4^. However, Si experiences a dramatic volume change (>300%) during the lithium alloying/dealloying processes, and for crystalline Si (c-Si) this large volume expansion is accompanied with dramatic anisotropic expansion^5^,^6^,^7^. This change not only causes severe pulverization of the material but also induces electrical disconnection of the active material from the current collector, resulting in performance degradation of the battery if Si is used as the anode. To minimize the extent of volume change, tremendous efforts have been made on the synthesis of novel nanostructured Si materials, such as nanowires^8^,^9^, nanotubes^10^,^11^,^12^, hollow spheres, and core-shell structures^13^,^14^. Recently, three-dimensional porous structured Si has attracted significant attention. The pre-formed nanopores in the Si can provide a large space to accommodate the volume expansion, and therefore help to maintain the structure integrity when lithium alloys with Si. Moreover, this three-dimensional porous structure provides large surface area of the material to be accessible to the electrolyte and thus a short diffusion length for lithium ions to transport from electrolyte to Si, which facilitates the lithium alloying/dealloying processes at high current rates^15^,^16^,^17^,^18^,^19^,^20^.

To understand the lithiation/delithiation process of Si, it is of importance to directly observe the structural and chemical evolution during the process and thus correlate with the battery properties. Over the past few years, tremendous progress has been made toward developing methodologies for *in situ* observation of structural and chemical evolution of electrodes used for LIBs. Among them, *in situ* transmission electron microscopy (TEM) has been particularly informative and has revealed important features of the lithiation/delithiation process of Si nanoparticles and nanowires on phase transition, structural evolution, and lithiation kinetics^6^,^7^,^21^,^22^,^23^,^24^,^25^,^26^,^27^,^28^. Specifically, both c-Si nanoparticles and nanowires are reported to transform to amorphous Li_x_Si (a-Li_x_Si) via electrochemical-driven solid-state amorphization. With further lithiation, a-Li_x_Si transforms to crystalline Li_15_Si_4_ (c-Li_15_Si_4_)^7^,^21^,^22^,^26^. The fracture behaviour of c-Si nanoparticles during the first lithiation is reported to be particle-size-dependent. The critical fracture diameter is 150 nm, below which cracks do not form, and above which surface cracking and particle fracture takes place upon lithiation^7^. In comparison, the critical fracture diameter of amorphous Si (a-Si) particles is reported to be up to 870 nm. In addition, the lithiation reaction velocity of a-Si is approximately constant and does not slow as in c-Si, which suggests different stress evolution during lithiation and implies that a-Si may be a more desirable active material than c-Si^27^. These studies have led to fundamental understanding of the lithiation/delithiation process of Si nanoparticles and nanowires; however, these studies cannot provide direct explanation of better electrochemical performance achieved by newly reported nanostructured Si than solid Si nanoparticles and nanowires. Moreover, most studies only focus on the first several lithiation/delithiation cycles of Si, but do not look into post-cycling analysis of the structural evolution of Si. In this work, we study the phase transition and structural evolution of both porous Si nanoparticles and porous Si nanowires by *in situ* and *ex situ* TEM. The *in situ* TEM observation of lithiation process of porous Si nanoparticles reveals that the lithiation proceeds in an end-to-end manner, which is different from the surface-to-center manner for solid Si nanoparticles under the same experimental condition. In addition, much larger critical fracture diameter is achieved in porous Si particle than previously reported for c-Si and a-Si particles. Another interesting feature in the lithiation process of porous Si nanoparticles and nanowires is that a-Li_x_Si does not transform to c-Li_15_Si_4_ even after full lithiation, which is distinct from that of solid Si nanoparticles and nanowires. The distinct lithiation behaviours of porous Si nanoparticles and nanowires are attributed to their interconnected three-dimensional porous structure, which is built up by numerous small domains. First-principle molecular dynamic simulation was conducted to investigate the effect of domain size on the phase stability of c-Li_15_Si_4_, which confirms the effect of nanostructure on phase transition. Moreover, structural evolution of porous and solid Si nanoparticles under successive lithiation/delithation cycles are compared through *ex situ* TEM, which confirms that porous Si is a more desirable anode material for LIBs than solid Si.

## Results

**Figure 1:**
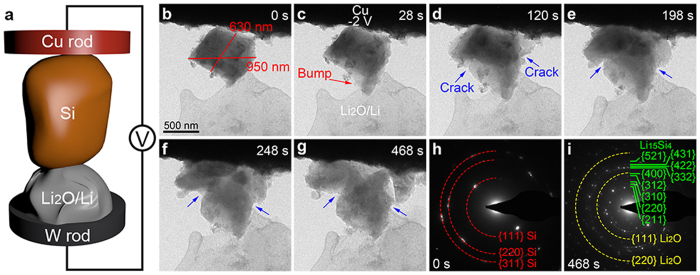
*In situ* TEM observation of the lithiation process of a ball-milled Si particle. The test was carried out using a nanobattery configuration with a ball-milled Si particle attached to a Cu rod as the working electrode, Li as the reference electrode, and Li_2_O as the solid electrolyte. (**a**) Schematic of the *in situ* TEM nanobattery. (**b**) TEM image of the ball-milled Si particle before lithiation. (**c**–**g**) Time series of the lithiation of the ball-milled Si particle, which illustrates the crack nucleation and fracture of the particle. After the Li_2_O/Li electrode contacted the ball-milled Si, a potential of −2 V was applied to the Cu electrode with respect to Li electrode to initiate the lithiation process. (**h**,**i**) Selected area electron diffraction (SAED) patterns of the ball-milled Si particle before (**h**) and after lithiation (**i**).

In this work, the porous Si nanoparticles and nanowires were prepared according to our previous reports^15^,^18^. To prepare porous Si nanoparticles, metallurgical Si was used as the starting material. After milling to submicron particles, the Si was etched in Fe(NO_3_)_3_/HF etchant to obtain porous structure. To prepare porous Si nanowires, a boron-doped Si wafer was used as the starting material and AgNO_3_/HF was used as the etchant to obtain the porous structure. To prepare Si nanowires without pores, a pure Si wafer without doping was used as the starting material. After etching by AgNO_3_/HF, Si nanowires without pores were obtained. For comparison, both Si nanoparticles with and without pores, and Si nanowires with and without pores are characterized by *in situ* TEM. The *in situ* TEM nanobattery setup is schematically shown in Fig. 1a. Figure 1b–g and Supplementary Movie 1 show the lithiation process of a ball-milled Si particle with largest diameter of ~950 nm and smallest diameter of ~630 nm. The ball-milled Si was prepared by ball-milling metallurgical Si and then being washed with HF and deionized water (DI-H_2_O) to remove the surface oxide layer (Supplementary Fig. 1a,b). After the Li_2_O/Li electrode contacted the ball-milled Si, a potential of −2 V was applied to the Cu electrode with respect to Li_2_O/Li electrode to initiate the lithiation process. As shown in Fig. 1c, a bump (indicated by the red arrow) comes out from the particle after lithiation for only 28 s, which is due to anisotropic expansion of Si particles. Further lithiation results in the change of contrast of the particle as shown in Fig. 1d. The gray Li_x_Si shell and dark Si core indicates that lithium ions flow from surface to center of the particle in the radial direction. As the particle size is well above the reported critical fracture diameter of c-Si (150 nm), cracks (indicated by the blue arrows) start to form in the particle after lithiation for only 120 s (Fig. 1d). After 468 s of lithiation, the particle fractures into several pieces (Fig. 1g). The selected area electron diffraction (SAED) pattern in Fig. 1h exhibits rings made up of discrete spots, indicating nanosized polycrystalline nature of the ball-milled Si particle before lithiation. The ball-milled Si particle is made up of nanosized single crystalline Si particles, which results in the anisotropic expansion of the ball-milled Si particle during lithiation process. After full lithiation, the particle transforms to polycrystalline Li_15_Si_4_ as indicated by Fig. 1i. The Li_2_O phase in Fig. 1i is from the Li_2_O/Li electrode in contact with the particle.

**Figure 2:**
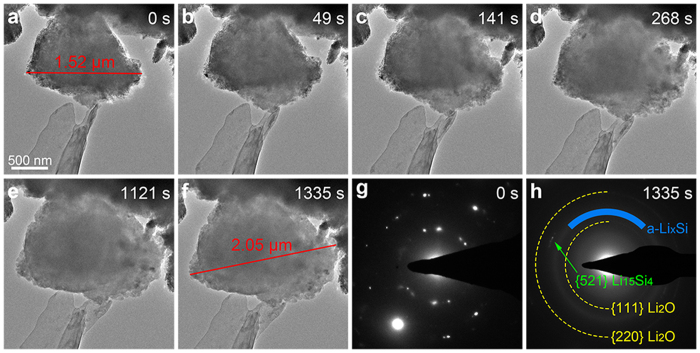
*In situ* TEM observation of the lithiation process of a porous Si particle. (**a**) TEM image of the porous Si particle with diameter up to 1.52 μm before lithiation. (**b**–**f**) Time series of the lithiation of the porous Si particle, which illustrates the volume expansion of the particle without crack formation. (**g**,**h**) SAED patterns of the porous Si particle before (**g**) and after lithiation (**h**).

Figure 2 shows the lithiation behaviour of a typical porous Si particle. As illustrated in Fig. 2a and Supplementary Fig. 1c,d, numerous pores distribute uniformly throughout the whole porous Si particle after electroless etching of the ball-milled Si. To investigate the fracture behaviour of porous Si particle during the lithiation process, we chose a large particle with diameter up to 1.52 μm for *in situ* TEM observation. Figure 2b–f demonstrate the TEM images of the porous Si particle during the lithiation process. From the TEM images and Supplementary Movie 2, the volume expansion of the particle initiates in the lower right corner and then proceeds to the top left corner of the particle. This indicates that the lithium ions flow in an end-to-end manner, which is distinct from the surface-to-center lithiation manner observed in both crystalline and amorphous Si particles^7^,^27^. To clarify the lithium propagation manner of porous Si particle, the lithiation behaviour of another porous Si particle was characterized by *in situ* TEM with higher magnification as shown in Supplementary Fig. 2a–d and Supplementary Movie 3. The lithiation front is marked by the red dotted line in Supplementary Fig. 2b–d, which propagates from lithium source to the other end of the particle. This observation is consistent with Fig. 2 and confirms the end-to-end lithiation manner of porous Si particle. After lithiation for 1121 s, the volume expansion of the particle almost ended (Fig. 2e). To ensure full lithiation of the particle, the −2 V potential was applied to the Cu electrode for another ~200 s and no obvious volume expansion of the particle was observed during this period. After lithiation for 1335 s, no crack was observed in the particle and the diameter of the particle increased to 2.05 μm, corresponding to a volume expansion of 145% (Fig. 2f). The volume expansion is far less than the theoretical 300% for solid Si particles after full lithiation. This is attributed to the porous structure of the particle, which provides large space to accommodate the volume expansion by possible inward expansion during the lithiation process. The SAED patterns of the particle were obtained before lithiation and after full lithiation as shown in Fig. 2g,h, respectively. Before lithiation, the porous Si particle is polycrystalline as shown in Fig. 2g. After full lithiation (Fig. 2h), the SAED pattern indicates that a-Li_x_Si (marked by the blue arc) and c-Li_15_Si_4_ (indicated by the green arrow) coexist^6^,^29^. The rings from Li_2_O/Li electrode are marked by the yellow arcs. This observation contrasts the SAED pattern of the fully lithiated ball-milled Si, which exhibits only c-Li_15_Si_4_ phase as shown in Fig. 1i. We note that the porous Si and ball-milled Si particles are prepared from the same starting material. Taking into account that the most distinguishable difference between the porous Si and the ball-milled Si is their microstructures, we believe that the porous structure helps to prevent the formation of c-Li_15_Si_4_ phase during the first lithiation process, and we will discuss it in detail later.

**Figure 3:**
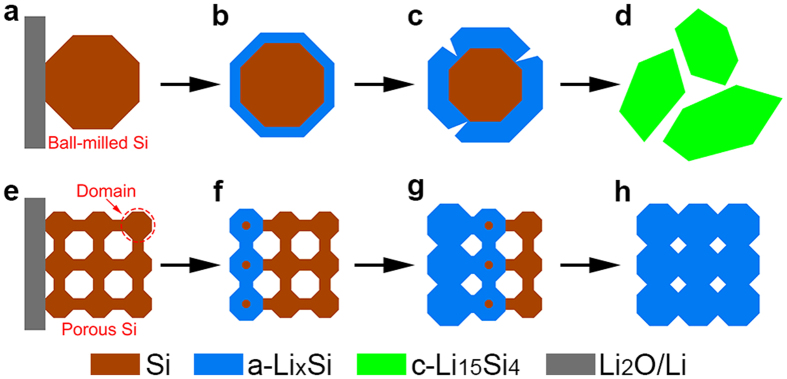
Schematic diagram illustrating the lithiation manners of ball-milled Si and porous Si nanoparticles. (**a**–**d**) Schematic diagram showing the surface-to-center lithiation manner of ball-milled Si particle. (**e**–**h**) Schematic diagram showing the end-to-end lithiation manner of porous Si particle.

A brief summary of the lithiation behaviours of solid and porous Si particles reveal that lithiation proceeds in a surface-to-center manner for solid Si particles while in an end-to-end manner for porous Si particles. Figure 3 schematically illustrates the different lithiation manners of solid and porous Si particles. As lithium diffuses faster in the surface of Si than that in the bulk, a-Li_x_Si shell will form in ball-milled Si particle once lithiation occurs (Fig. 3b). As the a-Li_x_Si shell thickens, cracks will form on the surface of the particle (Fig. 3c), which lead to final pulverization of the ball-milled Si particle as shown in Fig. 3d. The situation is different in porous Si particle, which is made up of numerous small domains (Fig. 3e) and possesses complex surface topological feature. The large and complex surface of porous Si lags the propagation of lithium in the whole particle. As a result, lithium tends to proceed from the lithium source and propagate through the whole particle in an end-to-end manner, even though lithium may proceed in a surface-to-center manner in each domain as shown in Fig. 3f,g. Because each domain in porous Si is in several nanometers, which is much smaller than the critical fracture diameter of solid Si, no crack will form during lithiation process (Fig. 3h). In addition, the porous structure provides large space to accommodate the volume expansion by possible inward expansion of each domain, leading to smaller volume change of the porous Si particle than solid Si particle.

**Figure 4:**
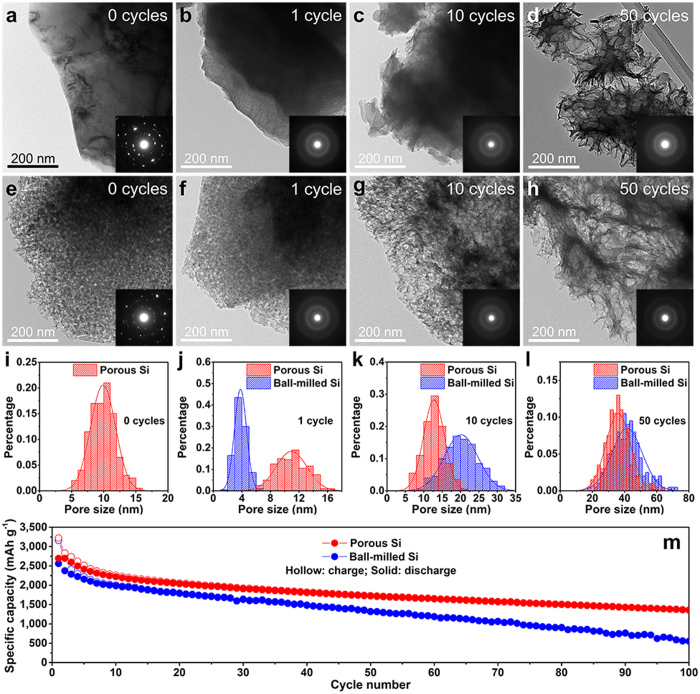
*Ex situ* TEM characterization of ball-milled Si and porous Si after different charge-discharge cycles and comparison of their cycling performances. The Si electrodes were cycled in Li-Si cells in the voltage window of 0.01–2 V (*vs.* Li/Li^+^) at a current density of 400 mA/g and then disassembled at the delithiated state before TEM observation. (**a–d**) TEM images of ball-milled Si before cycling (**a**), after cycling for 1 cycle (**b**), 10 cycles (**c**), and 50 cycles (**d**). (**e–h**) TEM images of porous Si before cycling (**e**), after cycling for 1 cycle (**f**), 10 cycles (**g**), and 50 cycles **(h**). The insets in (**a–h**) are the corresponding SAED patterns. (**i–l**) Pore size distributions of porous Si before cycling (**i**) and the comparison of ball-milled Si and porous Si after cycling for 1 cycle (**j**), 10 cycles (**k**), and 50 cycles (**l**). (**m**) Cycling performances of Li-Si cells using ball-milled Si and porous Si as working electrode, respectively. The galvanostatic charge-discharge test was carried out in the voltage window of 0.01–2 V (*vs.* Li/Li^+^) at a current density of 400 mA/g.

To further characterize the structural evolution of ball-milled Si and porous Si, *ex situ* TEM images and corresponding SAED patterns of the two samples were obtained before cycling and after being charge-discharged for 1 cycle, 10 cycles, and 50 cycles in Li-Si cells in the voltage window of 0.01–2 V (*vs.* Li/Li^+^) at a current density of 400 mA/g as shown in Fig. 4a–h. According to previous reports, the cutoff voltage range plays an important role to induce pore evolution in Si. Specifically, a large voltage window of 0.05-1 V would lead to porous structure of Si while Si cycled in a small voltage window of 0.17-0.6 V retains its original structure well after cycling^30^. In this work, we cycled the Li-Si cells in large voltage window of 0.01–2 V (*vs.* Li/Li^+^) so that we can study the capability of porous Si and ball-milled Si to suppress pore evolution during the cycling process. Before cycling, both ball-milled Si and porous Si are polycrystalline as indicated by the inset SAED patterns in Fig. 4a,e, respectively. After being charge-discharged for different cycles, both ball-milled Si and porous Si transform to amorphous structure as indicated by the inset SAED patterns in Fig. 4b–d,f–h. To quantitatively investigate the pore evolution processes of the two samples, pore size distributions were obtained based on statistical analysis of TEM images. Before cycling, the surface of ball-milled Si is smooth as shown in Fig. 4a. For porous Si particles, the pores are clearly resolved by the contrast in the image in Fig. 4e, and the mean diameter is 9.9 ± 0.1 nm based on the pore size distribution diagram in Fig. 4i. After being charge-discharged for only 1 cycle, nanopores are observed to form on the periphery of the ball-milled Si as indicated by the dark/light contrast in Fig. 4b, which is due to inelastic deformation of Li/Si during the lithiation/delithiation process^30^. On the contrary, the porous Si particle retains its original porous structure well as shown in Fig. 4f. This is confirmed by the pore size distribution diagram of two samples in Fig. 4j. The mean diameter of newly-formed pores in the ball-milled Si is 3.8 ± 0.1 nm. While for porous Si, the mean diameter of pores is 10.9 ± 0.1 nm, which is close to its original value before cycling. After cycling for 10 cycles, the surface of the ball-milled Si particles gets much rougher (Fig. 4c), while the pore size increase of porous Si is still not significant (Fig. 4g). As shown in Fig. 4k, the mean diameter of the pores in the ball-milled Si increases drastically to 20.1 ± 0.1 nm, corresponding to a 429% increase compared with that after 1 cycle. Similar to Ostwald ripening in which particles agglomerate to reduce surface energy, this increase of pore size with cycling is equivalent to agglomeration of pores so that the surface energy of the particle can be reduced^31^. In contrast to the significant pore size increase in ball-milled Si, the mean diameter of pores in porous Si is only 12.8 ± 0.1 nm after 10 charge-discharge cycles, corresponding to only 29% increase compared with that before cycling. After cycling for 50 cycles, pores in both ball-milled Si and porous Si increase in size as shown in Fig. 4d,h. According to Fig. 4l, the mean diameter of pores for ball-milled Si is 41.8 ± 0.1 nm. However, the mean diameter of pores for porous Si is 36.0 ± 0.1 nm, which is still smaller than that of ball-milled Si. As the pore evolution is due to inelastic deformation of Li/Si during lithiation/delithiation process, for solid Si nanoparticles, the inelastic deformation is severe due to its large volume change during lithiation/delithiation process. For porous Si nanoparticles, however, the domains of the particle are observed to expand into the void space in the particle based on the observation in Supplementary Fig. 2a–d that the contrast of the particle from the porous structure becomes obscure and uniform during the lithiation process. This lithiation behaviour of porous Si particle results in smaller volume change of the particle and stress relaxation in each domain. The stress relaxation prevents the stress in porous Si nanostructures from exceeding the elastic limit of Si, and thus suppresses the pore evolution in porous Si nanostructures.

The formation and size increase of pores in ball-milled Si particles would cause significant volume expansion of particles as they transform from solid particles to totally porous structure. However, with pre-formed pores, the volume change of porous Si particles before and after cycling is much less significant than that of ball-milled Si particles. This difference in particle volume change during cycling results in the different cycling performance of the two electrodes. Figure 4m shows the cycling performances of ball-milled Si and porous Si electrodes tested in the voltage window of 0.01–2 V (*vs.* Li/Li^+^) at a current density of 400 mA/g. As shown in the figure, the capacity of ball-milled Si decays rapidly in the initial 10 cycles and then decreases in constant rate. This corresponds to the TEM observation that pore formation in ball-milled Si particles takes place in early cycles, which causes the particles to lose electrical contact from the current collector and thus leads to loss of active materials for capacity contribution. The volume change of porous Si during cycling is much less than that of ball-milled Si; however, the relatively large surface area of porous Si as compared to ball-milled Si would lead to more severe solid electrolyte interface (SEI) formation on porous Si particles, which would also cause capacity decay in the initial cycles. For this reason, it is essential to apply coating on porous Si particles (*e.g.* carbon coating) to mitigate the SEI formation and thus to further improve the cyclability of porous Si electrodes^17^,^18^,^19^.

**Figure 5:**
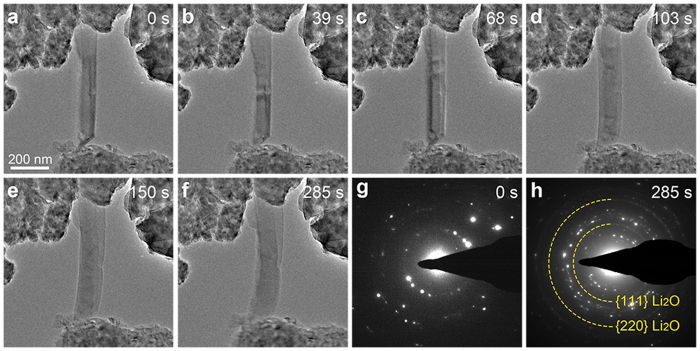
*In situ* TEM observation of the lithiation process of a typical Si nanowire. (**a**) TEM image of the Si nanowire before lithiation. (**b**–**f**) Time series of the lithiation of the Si nanowire. (**g**,**h**) SAED patterns of the Si nanowire before (**g**) and after lithiation (**h**).

In order to further demonstrate whether the microstructure or the starting material of porous Si would affect its lithiation behaviour, we prepared solid Si nanowires and porous Si nanowires according to our previous report using Si wafers as the starting material^15^. Figure 5 and Supplementary Movie 4 show the lithiation behaviour of a typical solid Si nanowire with diameter of ~120 nm and length of ~600 nm. The Si nanowire before lithiation is shown in Fig. 5a and Supplementary Fig. 3a,b. After lithiation for 103 s (Fig. 5d), the gray shell and dark core of the nanowire reveal that the lithiation of Si nanowire occurs through the formation of a-Li_x_Si shell and Si core structure, which is due to the faster lithium diffusion rate on the nanowire surface than that in the center. After lithiation for 150 s, the volume expansion of the nanowire almost ended (Fig. 5e). To ensure full lithiation of the nanowire, the −2 V potential was applied to the Cu electrode for another ~130 s and no obvious volume expansion of the nanowire was observed during this period. After lithiation for 285 s, no crack was observed in the nanowire (Fig. 5f). This is in agreement with a previous report, which demonstrates that the critical diameter for pulverization of Si nanowire is in the regime of 220–260 nm^32^. The SAED pattern of the Si nanowire before lithiation (Fig. 5g) reveals its polycrystalline nature. After lithiation for 285 s, the SAED pattern of the nanowire (Fig. 5h) indicates that it has transformed to the c-Li_15_Si_4_ phase, which is similar to the result of the ball-milled Si particle in Fig. 1i.

**Figure 6:**
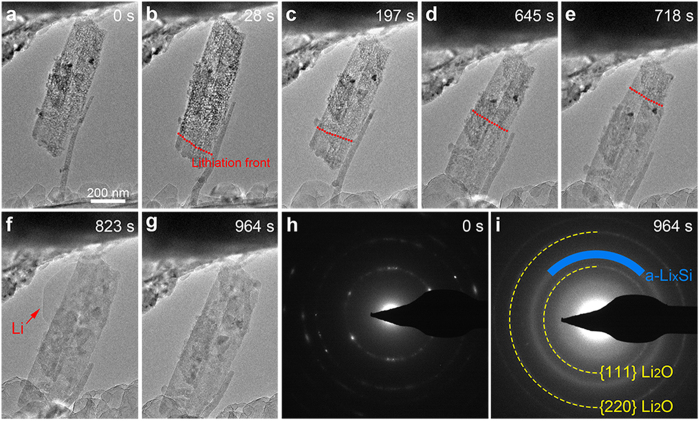
*In situ* TEM observation of the lithiation process of a porous Si nanowire bundle. (**a**) TEM image of the porous Si nanowire bundle before lithiation. (**b**–**g**) Time series of the lithiation of the porous Si nanowire bundle. The single nanowire beside the bundle provides lithium diffusion path. (**h**,**i**) SAED patterns of the Si nanowire bundle before (**h**) and after lithiation (**i**).

The lithiation behaviour of a porous Si nanowire bundle consisting of several porous Si nanowires was also examined by *in situ* TEM as demonstrated in Fig. 6 and Supplementary Movie 5. As shown in Fig. 6a and Supplementary Fig. 3c,d, the nanowires obtain highly porous structure with pore diameter and wall thickness of ~8 nm before lithiation. The single nanowire beside the nanowire bundle in Fig. 6a acts as the lithium diffusion path during the lithiation process. Figure 6b–g demonstrate the lithiation process of the porous Si nanowire bundle, from which we can find that the contrast of the nanowires from the porous structure becomes obscure and uniform during the process. This indicates that the a-Li_x_Si expands into the void space in the nanowires, which helps to minimize the volume expansion of the nanowires. The lithiation front is marked by the red dotted line in Fig. 6b–e, which also indicates the end-to-end lithiation manner similar to that of porous Si particle. This observation demonstrates that the explanation of porous Si particle lithiation manner in Fig. 3 also applies to porous Si nanowires. After lithiation for 823 s, lithium was observed to diffuse out of the nanowire bundle as indicated by the red arrow in Fig. 6f, indicating that the lithiation process was complete. To ensure full lithiation, the −2 V potential was applied to the Cu electrode for another ~140 s and no obvious volume expansion of the nanowire bundle was observed during this period. After lithiation for 964 s, no crack was observed in the nanowire bundle (Fig. 6g). Figure 6h shows the SAED pattern of the nanowire bundle before lithiation, which reveals its polycrystalline nature. After lithiation for 964 s, the SAED pattern of the porous Si nanowire bundle (Fig. 6i) demonstrates that it has transformed to a-Li_x_Si (marked by the blue arc). This observation contrasts the SAED pattern of the fully lithiated solid Si nanowire, which exhibits only c-Li_15_Si_4_ phase as shown in Fig. 5h.

A brief summary of the lithiation behaviours of porous Si and solid Si nanostructures reveal that after full lithiation, solid Si nanostructures transform to c-Li_15_Si_4_ while porous Si nanostructures transform to a-Li_x_Si. The porous Si nanostructures are made up of small Si domains as shown in Supplementary Figs 1d and 3d, while the domain of solid Si nanoparticle or solid Si nanowire is the whole nanoparticle or whole nanowire due to their solid structures. We believe that the different sizes of the domains of porous Si and solid Si lead to their different phase transition behaviours. To further illustrate the effect of domain size on the resultant phase after lithiation, first-principle molecular dynamic simulation was performed to study the structure stability of nanosized c-Li_15_Si_4_ particle. The simulated nanoparticle was constructed by 2 × 2 × 2 Li_15_Si_4_ crystalline supercells, which is composed of 128 Si atoms and 480 Li atoms, and corresponds to the size of 2 nm in three dimensions. Periodic boundary condition is applied in the simulation, and the empty space between Li_15_Si_4_ particles is set larger than 1 nm to exclude the mutual interaction of atoms from neighbouring particles. First-principle calculations were performed using the VASP code density functional theory (DFT) calculations in generalized gradient approximation (GGA) with the Perdew-Burke-Ernzerhof (PBE) function used to calculate the force among atoms^33^,^34^. Molecular dynamic simulation was carried out at 300 K with a time step of 1 fs interval.

**Figure 7:**
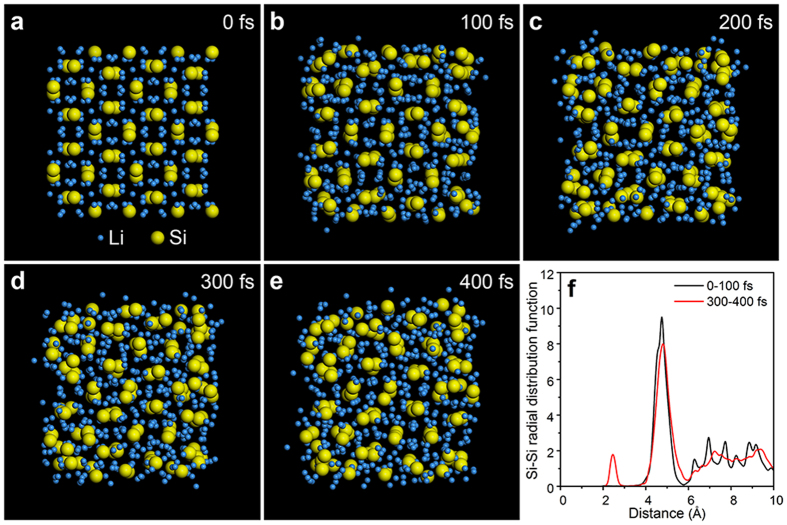
First-principle molecular dynamic simulation to study the structure stability of a nanosized c-Li_15_Si_4_ particle. (**a**) The modeled structure of c-Li_15_Si_4_. (**b**–**e**) Atomic structure and morphology of the Li_15_Si_4_ particle at different simulation stages. (**f**) Si-Si radial pair distribution function at different stages of the simulated process. The appearance and increasing intensity of the peak at 2.5 Å indicate the intermixing of Si and Li to form an amorphous phase.

Figure 7a–e show the structural evolution of Li_15_Si_4_ nanoparticle from the initial crystal to a disordered structure after 400 fs simulation. The yellow atoms are Si, and blue atoms are Li. At the early stage of the simulation (*e.g.* 100 fs), it is clear to see that the surface atoms are the first to deviate from their original positions due to the lack of symmetric force potential at the particle surface (Fig. 7b). In the following simulation, cascaded breakdown of the periodic force potential leads to the structure disordering from outer surface to the inner part of particle. After 400 fs simulation, the particle turns to an amorphous structure (Fig. 7e). To semi-quantify the structure amorphization, the radial distribution function (RDF) of Si-Si pairs was calculated and shown in Fig. 7f. At the initial stage (0–100 fs), the sharp peaks in RDF illustrate the well-defined crystal structure. However, after 400 fs simulation, peaks at large Si-Si distance are largely smoothed, indicating the disappearance of ordered atomic arrangement. The small peak showing up at 2.5 Å corresponds to the distance of Si-Si in the amorphous Si structure, which further demonstrates the destroying of crystalline Li_15_Si_4_ structure.

Due to the constrained computation resource for first-principle molecular dynamic simulation of large-size particles, we adopted classical molecular dynamic simulation to characterize the structure stability of c-Li_15_Si_4_ particles with the same initial crystal structure as 2 nm particle (Fig. 7a) while with larger diameter of 6 nm, 8 nm, 10 nm, and 12 nm. After 400 fs simulation, the atomic structures and morphologies of the Li_15_Si_4_ particles are illustrated in Supplementary Fig. 4. The yellow atoms are Si, and blue atoms are Li. Periodic boundary condition was used with particle-to-particle distance larger than 5 nm to eliminate the mutual interaction. Simulation were conducted by using the LAMMPS software code^35^, and a second nearest neighbour (2NN) modified embedded atom method (MEAM) potential was used to account for the atomic interaction in Li-Si system^36^. Based on the comparison of enlarged images in Supplementary Fig. 4a–d, it is found that in 6 nm Li_15_Si_4_ particle (Supplementary Fig. 4a), the surface of the particle is in amorphous structure and the core atoms have lost their initial crystalline arrangement. In 8 nm particle, however, the crystallinity of the core increases compared with that of 6 nm particle even though the surface atoms in the 8 nm particle still rearrange in amorphous structure (Supplementary Fig. 4b). Similar trend is observed in 10 nm particle (Supplementary Fig. 4c) and when particle size increases to 12 nm, the crystallinity of the core is the highest and the crystalline volume is the largest among four particles even though the surface of 12 nm particle still tends to be amorphous (Supplementary Fig. 4d). Generally speaking, the trend is that as the particle size increases, the crystallinity in the core of the particles and the crystalline volume in the particles also increase. However, due to lack of symmetric force potential in the surface, the surface atoms in the particles always tend to deviate from their original positions and thus rearrange in amorphous structure. This simulation result further supports our conclusion that the small domains in porous Si nanostructures help to suppress c-Li_15_Si_4_ formation during the first lithiation process. Besides, this explains why some diffraction spots of c-Li_15_Si_4_ show up in Fig. 2h, which may be due to the formation of c-Li_15_Si_4_ in the cores of some large-size domains in the porous Si particle after full lithiation. For porous Si nanowire, however, only a-Li_x_Si forms after full lithiation (Fig. 6i), which is possibly due to the smaller size of domains in porous Si nanowires than that in porous Si nanoparticles as we compare the domains marked in Supplementary Figs 1d and 3d. The formation of c-Li_15_Si_4_ during the first lithiation process is reported to be detrimental to the cycle life of Si-based LIBs and a cutoff voltage higher than 0.05 V is usually selected to suppress the formation of c-Li_15_Si_4_ at low potential^37^. Here, we report that in addition to the low cutoff voltage, the nanoporous structure can also suppress the formation of c-Li_15_Si_4_ during first lithiation process due to the effect of domain size, which helps to achieve the excellent cycling performances of porous Si nanostructures.

## Conclusion

In conclusion, we have applied *in situ* and *ex situ* TEM to study the structural evolution and phase transition of porous Si nanoparticles and nanowires and have compared their behaviours with solid Si nanoparticles and nanowires. The critical fracture diameter of porous Si particles reaches up to 1.52 μm, which reveals its better capacity to accommodate volume expansion during the lithiation process. In addition, the porous Si nanoparticles and nanowires transform to the a-Li_x_Si phase after full lithiation in contrast to the c-Li_15_Si_4_ phase for solid Si nanoparticles and nanowires, which is due to small Si domains in porous Si nanoparticles and nanowires as revealed by the first-principle molecular dynamic simulation. Finally, *ex situ* TEM observation of porous Si nanoparticles and solid Si nanoparticles reveal that porous Si nanoparticles obtain better capability to suppress pore evolution than solid Si nanoparticles during the cycling process. The better capabilities of porous Si nanostructures to accommodate volume expansion, to suppress c-Li_15_Si_4_ formation during the first lithiation process, and to suppress pore evolution during cycling make them more desirable lithium-ion battery anode materials than solid Si nanostructures.

## Methods

### Materials preparation

Synthesis of porous Si particles: Porous Si particles were synthesized according to our previous report^18^. Specifically, metallurgical Si particles were ground to fine powder using ball-milling operated at grinding speed of 1200 rpm for 5 hours. After that, the Si particles were soaked in a ferric etchant containing 0.03 M Fe(NO_3_)_3_ and 5 M HF under continuous stirring for 2 hours. The precipitates containing porous Si particles were then collected and washed with ethanol and DI-H_2_O. After drying at 90 °C in air for 6 hours, the particles were collected for further use.

Synthesis of ball-milled Si particles: Metallurgical Si particles were ground to fine powder using ball-milling operated at grinding speed of 1200 rpm for 5 hours. The Si powder was then washed with HF and DI-H_2_O successively to remove surface oxide layer. After drying at 90 °C in air for 6 hours, the particles were collected for further use.

Synthesis of porous Si nanowires: Porous Si nanowires were synthesized according to our previous report^15^. Specifically, boron-doped Si wafers (resistivity <5 mΩ·cm) were immersed in an etchant solution containing 0.02 M AgNO_3_ and 5 M HF for 3 h. After being washed with DI-H_2_O, concentrated HNO_3_, and DI-H_2_O again, sequentially, porous Si nanowires were collected by scratching the wafers using a blade.

Synthesis of solid Si nanowires: Si wafers without doping were immersed in an etchant solution containing 0.02 M AgNO_3_ and 5 M HF for 3 h. After being washed with DI-H_2_O, concentrated HNO_3_, and DI-H_2_O again, sequentially, solid Si nanowires were collected by scratching the wafers using a blade.

Preparation of Si-based electrodes: The active material can be either porous Si nanoparticles or ball-milled Si nanoparticles. To prepare electrodes, active Si material was first mixed with carbon black and alginic acid sodium salt with mass ratio of 7:2:1 in water to form uniform slurry. The slurry was coated on copper foil and then dried at 90 °C in air for 6 hours.

### Electrochemical measurements

For battery measurements, CR2032 coin cells were assembled using lithium foil as counter/reference electrode and Celgard 2400 as separator. The prepared Si-based electrodes were used as working electrodes. The electrolyte was 1 M LiPF_6_ in dimethyl carbonate (DMC)/fluoroethylene carbonate (FEC), 1:1 by volume. The galvanostatic charge-discharge test was carried out in the voltage window of 0.01–2 V (*vs.* Li/Li^+^) at a current density of 400 mA/g.

### *In situ* TEM characterization

The experimental setup is schematically illustrated in Fig. 1a. The *in situ* TEM characterization was conducted using a nanobattery configuration with Si (ball-milled Si nanoparticles, porous Si nanoparticles, solid Si nanowires, or porous Si nanowires) as the working electrodes, Li as the reference electrode, and Li_2_O as the solid electrolyte. All the *in situ* electrochemical tests were conducted in a Titan 80–300 scanning transmission electron microscope (STEM) operated at 300 kV with a Nanofactory TEM scanning tunneling microscopy (STM) holder. To assemble the nanobattery, a Cu rod and a W rod were firstly cut to produce clean and fresh cross section. Si was randomly attached to Cu rod as the working electrode by directly touching Si powder with the Cu rod. After that, the holder was transferred to Ar-filled glovebox, in which the W rod was used to scratch the Li metal surface to fetch some fresh Li. A conformal coating layer of Li on one end of the W rod served as the reference electrode and lithium source. The W rod was then mounted onto the holder by a screw for reliable mechanical and electrical connection. The entire assembly was then transferred to the microscope column within a sealed plastic bag. The lithium metal was only exposed to air during the insertion of the TEM holder into the microscope column, which is typically about 2 s. During this short period of air exposure, the surface of the lithium metal was oxidized to Li_2_O, which acts as the solid electrolyte for the function of the nanobattery. To initiate lithiation process, a bias of −2 V was applied on the Si electrode against the Li electrode to promote Li ion transport through the Li_2_O layer.

### *Ex situ* TEM characterization

To characterize the structural evolution of ball-milled Si nanoparticles and porous Si nanoparticles after different charge-discharge cycles, the Li-Si cells were cycled in the voltage window of 0.01–2 V (*vs.* Li/Li^+^) at a current density of 400 mA/g, and were then disassembled at delithiated state after different cycles inside Ar-filled glovebox. The active materials from the electrodes were then washed with acetonitrile and 0.5 M H_2_SO_4_ to remove the residual electrolyte and lithium salts. After that, the active materials were washed with DI-H_2_O and ethanol, and finally dried at 90 °C in air for 6 hours before *ex situ* TEM characterization. Transmission electron microscope (JEOL, JEM-2100F) was used for *ex situ* TEM characterization in the paper. The pore size distributions were obtained based on statistical analysis of TEM images. For each sample, 200 pores were selected from TEM images and the largest diameter of each pore was measured for statistical analysis.

## Additional Information

**How to cite this article**: Shen, C. *et al.* *In Situ* and *Ex Situ* TEM Study of Lithiation Behaviours of Porous Silicon Nanostructures. *Sci. Rep.* **6**, 31334; doi: 10.1038/srep31334 (2016).

## Supplementary Material

Supplementary Information

Supplementary Movie 1

Supplementary Movie 2

Supplementary Movie 3

Supplementary Movie 4

Supplementary Movie 5
