# Supplementary material for: In Situ and Ex Situ TEM Study of Lithiation Behaviours of Porous Silicon Nanostructures
Source: Sci Rep. 2016 Aug 30;6:31334. doi: 10.1038/srep31334 (PMC5004143; doi:10.1038/srep31334)
Supplement: Supplementary Information [file srep31334-s1.doc]

***In Situ* and *Ex Situ* TEM Study of Lithiation Behaviours of Porous Silicon Nanostructures**

Chenfei Shen^1,†^, Mingyuan Ge^1,2,†^, Langli Luo^3^, Xin Fang^1^, Yihang Liu^4^, Anyi Zhang^1^, Jiepeng Rong^1^, Chongmin Wang^3^, Chongwu Zhou^1,4,*^

^1^Mork Family Department of Chemical Engineering and Materials Science, University of Southern California, Los Angeles, California 90089, United States.

^2^National Synchrotron Light Source II, Brookhaven National Laboratory, Upton, New York 11973, United States.

^3^Environmental Molecular Sciences Laboratory, Pacific Northwest National Laboratory, Richland, Washington 99352, United States.

^4^Ming Hsieh Department of Electrical Engineering, University of Southern California, Los Angeles, California 90089, United States.

^*^Corresponding author.

Correspondence and requests for materials should be addressed to C.Z. (chongwuz@usc.edu).

^†^These authors contributed equally to this work.

**Supplementary Figures:**

**
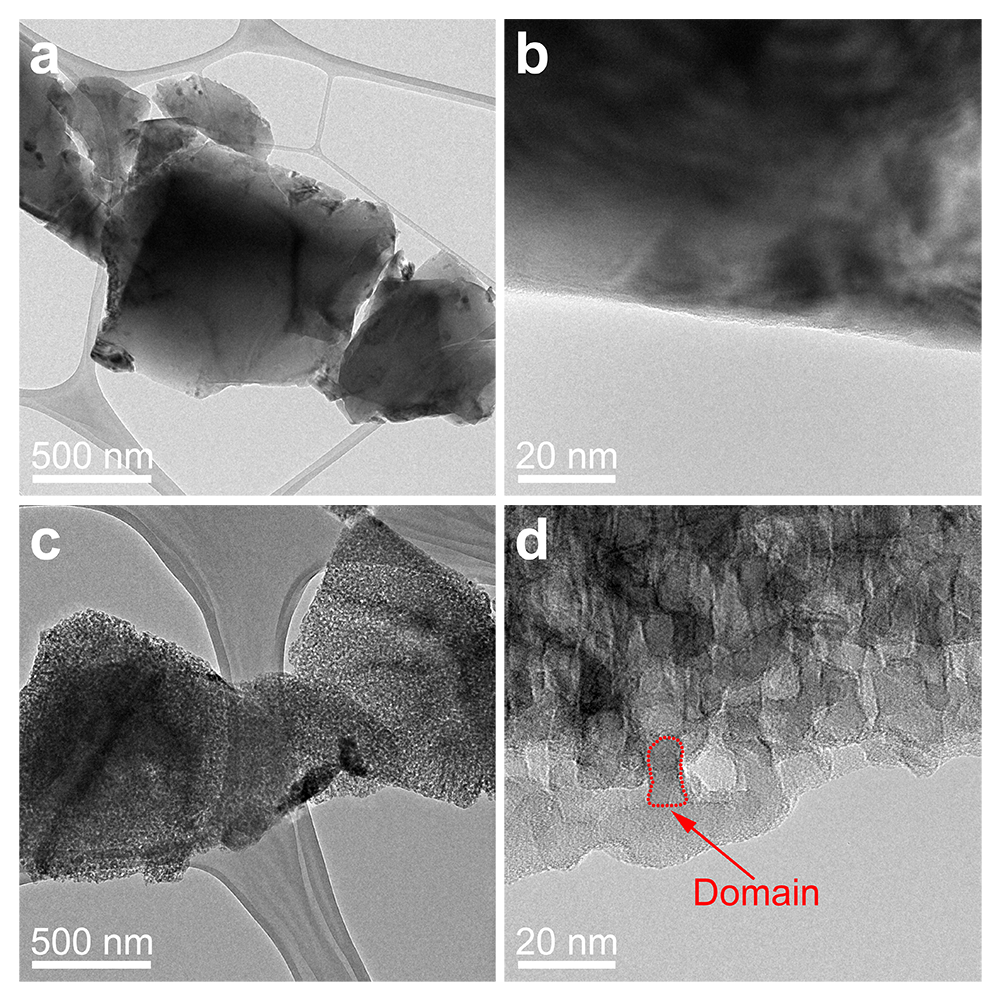
**

**Supplementary Figure 1. Characterization of ball-milled Si nanoparticles and porous Si nanoparticles.** (**a**,**b**) TEM images of ball-milled Si nanoparticles at different magnifications. (**c**,**d**) TEM images of porous Si nanoparticles at different magnifications. One domain of the porous Si nanoparticle was marked by the red dotted line in (**d**).


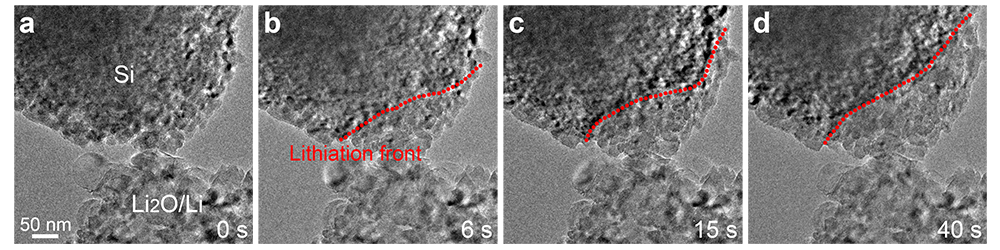


**Supplementary Figure 2. *In situ* TEM observation of the lithiation process of a porous Si particle with high magnification to show the lithiation front.** (**a**) TEM image of the porous Si particle before lithiation. (**b**-**d**) Time series of the lithiation of the porous Si particle showing the propagation of the lithiation front, which is indicated by the red dotted line.

**
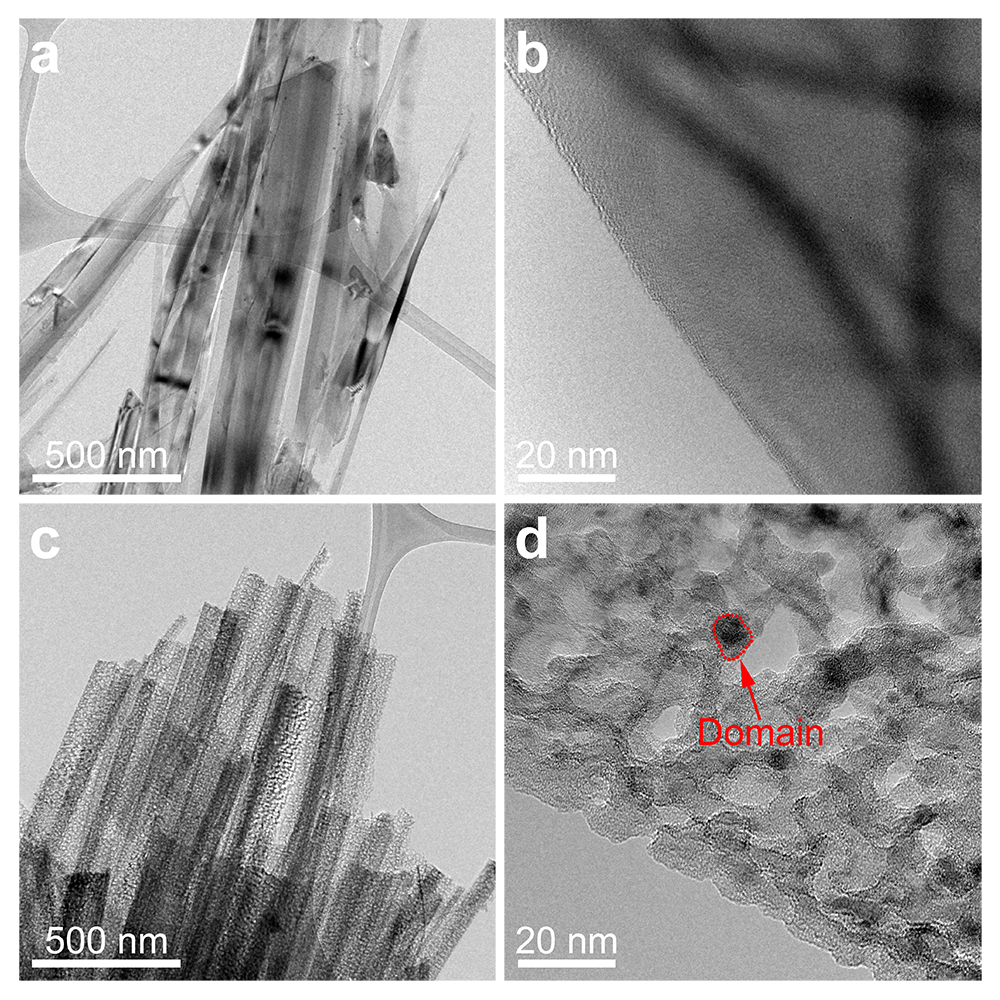
**

**Supplementary Figure 3. Characterization of solid Si nanowires and porous Si nanowires.** (**a**,**b**) TEM images of solid Si nanowires at different magnifications. (**c**,**d**) TEM images of porous Si nanowires at different magnifications. One domain of the porous Si nanowire was marked by the red dotted line in (**d**).


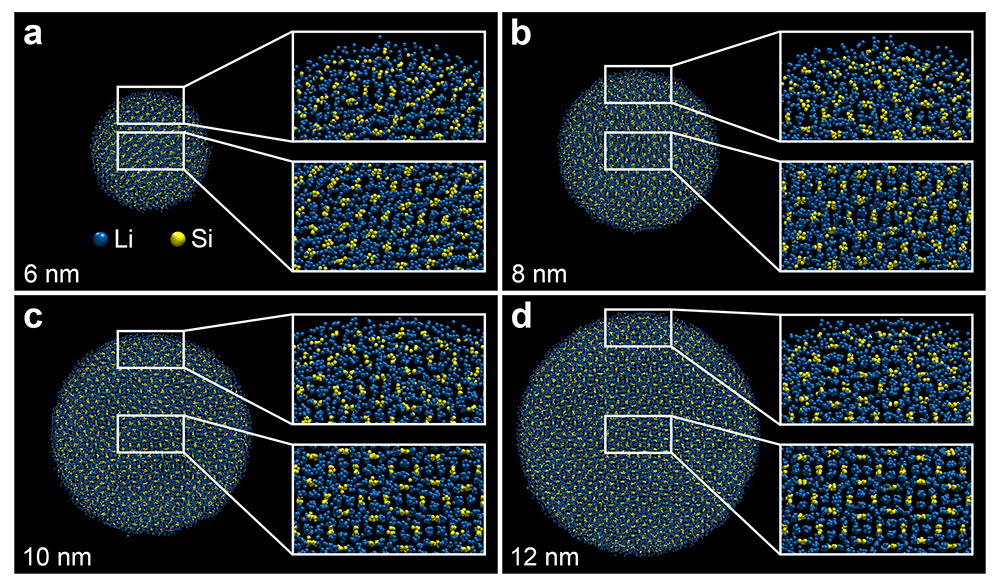


**Supplementary Figure 4. Classical molecular dynamic simulation to study the structure stability of c-Li_15_Si_4_ particles with different sizes.** Atomic structures and morphologies of the Li_15_Si_4_ particles with diameter of 6 nm (**a**), 8 nm (**b**), 10 nm (**c**), and 12 nm (**d**) after 400 fs simulation. The insets in (**a**-**d**) are the enlarged images showing the surface and core of the corresponding particles.

**Supplementary Movies:**

**Supplementary Movie 1.** *In situ* TEM movie showing the lithiation of the ball-milled Si particle shown in Fig. 1 in the main text. This movie is presented at 32 times actual speed.

**Supplementary Movie 2.** *In situ* TEM movie showing the lithiation of the porous Si particle shown in Fig. 2 in the main text. This movie is presented at 64 times actual speed.

**Supplementary Movie 3.** *In situ* TEM movie showing the lithiation of the porous Si particle shown in Supplementary Fig. 2 in the Supplementary Information. This movie is presented at 4 times actual speed.

**Supplementary Movie 4.** *In situ* TEM movie showing the lithiation of the solid Si nanowire shown in Fig. 5 in the main text. This movie is presented at 16 times actual speed.

**Supplementary Movie 5.** *In situ* TEM movie showing the lithiation of the porous Si nanowire bundle shown in Fig. 6 in the main text. This movie is presented at 64 times actual speed.
